# The landscape of Mucopolysaccharidosis in Southern and Eastern European countries: a survey from 19 specialistic centers

**DOI:** 10.1186/s13023-022-02285-x

**Published:** 2022-03-24

**Authors:** Anna Tylki-Szymańska, Zsuzsanna Almássy, Violetta Christophidou-Anastasiadou, Daniela Avdjieva-Tzavella, Ingeborg Barisic, Rimante Cerkauskiene, Goran Cuturilo, Maja Djiordjevic, Zoran Gucev, Anna Hlavata, Beata Kieć-Wilk, Martin Magner, Ivan Pecin, Vasilica Plaiasu, Mira Samardzic, Dimitrios Zafeiriou, Ioannis Zaganas, Christina Lampe

**Affiliations:** 1 Department of Pediatric Nutrition and Metabolic Diseases, The Children’s Memorial Health Institute, Warsaw, Poland; 2 Department of Toxicology and Metabolic Diseases, Heim Pal Children’s Hospital Budapest, Budapest, Hungary; 3 Archbishop Makarios III Hospital, Nicosia, Cyprus; 4 Department of Clinical Genetics, University Pediatric Hospital, Sofia, Bulgaria; 5 Centre of Excellence for Reproductive and Regenerative Medicine, Children’s Hospital Zagreb, Medical School University of Zagreb, Zagreb, Croatia; 6 Clinic of Paediatrics, Institute of Clinical Medicine, Faculty of Medicine, Vilnius University, Vilnius, Lithuania; 7 Faculty of Medicine, University of Belgrade, Belgrade, Serbia; 8 University Children’s Hospital, Belgrade, Serbia; 9 Mother and Child Health Care Institute of Serbia, Medical University of Belgrade, Belgrade, Serbia; 10 University Children’s Hospital, Skopje, North Macedonia; 11 National Institute of Children’s Diseases, Department of Paediatrics, Medical Faculty Comenius University, Centre for Inherited Metabolic Disorders, Bratislava, Slovakia; 12 Unit of Rare Metabolic Diseases, Department of Metabolic Diseases, Jagiellonian University Medical College, University Hospital, Krakow, Poland; 13 Department of Paediatrics, University Thomayer Hospital and First Faculty of Medicine, Charles University, Prague, Czech Republic; 14 Department of Pediatrics, General University Hospital and First Faculty of Medicine, Charles University, Prague, Czech Republic; 15 University Hospital Centre Zagreb, Department of Internal Medicine, Division of Metabolic Diseases, Zagreb School of Medicine, Zagreb, Croatia; 16 Regional Centre of Medical Genetics, INSMC Alessandrescu-Rusescu, Bucharest, Romania; 17 Institute for Sick Children, Department of Pediatric Endocrinology and Metabolism, Medical School, University of Montenegro, Podgorica, Montenegro; 18 First Department of Pediatrics, Hippokratio General Hospital, Aristotle University, Thessaloniki, Greece; 19 Neurogenetics Laboratory, Neurology Department, University Hospital of Heraklion, University of Crete, Heraklion, Greece; 20 Department of Child Neurology, Epileptology and Social Pediatrics, Centre for Rare Diseases, University of Giessen, Standort Giessen, Feulgenstr. 12, 35389 Giessen, Germany

**Keywords:** Mucopolysaccharidoses, Morquio A syndrome, Southern and Eastern European countries, Enzyme replacement therapy, Treatment accessibility

## Abstract

**Background:**

Mucopolysaccharidoses (MPS) are a group of lysosomal storage disorders caused by defects in genes coding for different lysosomal enzymes which degrade glycosaminoglycans. Impaired lysosomal degradation causes cell dysfunction leading to progressive multiorgan involvement, disabling consequences and poor life expectancy. Enzyme replacement therapy (ERT) is now available for most MPS types, offering beneficial effects on disease progression and improving quality of life of patients. The landscape of MPS in Europe is not completely described and studies on availability of treatment show that ERT is not adequately implemented, particularly in Southern and Eastern Europe. In this study we performed a survey analysis in main specialist centers in Southern and Eastern European countries, to outline the picture of disease management in the region and understand ERT implementation. Since the considerable number of MPS IVA patients in the region, particularly adults, the study mainly focused on MPS IVA management and treatment.

**Results:**

19 experts from 14 Southern and Eastern European countries in total responded to the survey. Results outlined a picture of MPS management in the region, with a high number of MPS patients managed in the centers and a high level of care. MPS II was the most prevalent followed by MPS IVA, with a particular high number of adult patients. The study particularly focused on management and treatment of MPS IVA patients. Adherence to current European Guidelines for follow-up of MPS IVA patients is generally adequate, although some important assessments are reported as difficult due to the lack of MPS skilled specialists. Availability of ERT in Southern and Eastern European countries is generally in line with other European regions, even though regulatory, organizational and reimbursement constrains are demanding.

**Conclusions:**

The landscape of MPS in Southern and Eastern European countries is generally comparable to that of other European regions, regarding epidemiology, treatment accessibility and follow up difficulties. However, issues limiting ERT availability and reimbursement should be simplified, to start treatment as early as possible and make it available for more patients. Besides, educational programs dedicated to specialists should be implemented, particularly for pediatricians, clinical geneticists, surgeons, anesthesiologists and neurologists.

## Background

The mucopolysaccharidoses (MPS) are a group of progressive inherited metabolic disorders caused by defects in the lysosomal enzymes required for the degradation of glycosaminoglycans (GAG). As a result of impaired catabolism, GAGs accumulate in lysosomes, causing cell dysfunction that leads to multisystemic clinical manifestations. Depending on the lysosomal enzyme involved, catabolism of single or multiple GAGs may be blocked, leading to distinct phenotypes classified as different types of MPS. Eleven enzymatic defects are described, causing seven different MPS types, with several subtypes [1]. All MPS disorders share similar clinical multisystemic manifestations, including *dysostosis multiplex*, short stature, coarse facial features, kyphoscoliosis and spinal degenerative changes, subluxations or stenosis with compression of nerves or the spinal cord, joint stiffness, cognitive dysfunction, hepatosplenomegaly and hernias. Hearing, vision, and cardiopulmonary functions are also affected. The skeletal disorder is the most common symptom across MPS types, with frequent loss of ambulation, chronic inflammation and disabling pain in the joints. As a result, patients with MPS often have a low quality of life, a short life expectancy and require life-long treatment [2, 3]. Historically, treatment of MPS has been symptomatic, mainly employing physical rehabilitation and surgery to alleviate the burden of skeletal deterioration. The first etiological treatment was allogenic transplant of hematopoietic cells (HSCT) in MPS I patients in 1980 [4]. At present, HSCT is an established treatment only for MPS I. However, specific enzyme replacement therapies (ERT) are now available for five MPS types—MPS I, II, IVA, VI and VII—based on human recombinant enzymes to replace the defective lysosomal enzymatic function. ERT is effective in alleviating the somatic clinical manifestations of the various MPS, including reduction of respiratory dysfunction, hepatomegaly and joint stiffness, significantly improving quality of life for patients with MPS. However, treatment efficacy remains sub-optimal, since several established symptoms and complications cannot be reversed [3].

Importantly, these novel treatments led to increased life expectancy, necessitating management through transition from childhood to adulthood for more patients. As for all rare metabolic diseases, MPS patients receive adequate pediatric care, while a structured transition process for patients going through adolescence, and then adulthood, is not always implemented [5].

In Europe, the landscape of MPS epidemiology and management, including implementation of transition of care and ERT availability, is not completely defined, particularly for Southern and Eastern countries [2]. With this publication, we aim to describe the real life MPS management in Southern and Eastern European countries. The survey revealed a high number of MPS IVA patients in the region, the second largest after MPS II and, most notably, the highest number of adult patients. These data, together with the availability of recent European guidelines for management and treatment of MPS IVA patients, led us to particularly focus on MPS IVA management and treatment in the region. Moreover, despite established clinical guidelines, as it often occurs for rare diseases, the small number of studies on MPS IVA patients is a main obstacle for clinicians on the way to standard clinical practice. Also, considering the high number of adult patients with MPS IV A in Southern and Eastern European countries, transition to adult care and treatment availability for adult patients are particularly concerning.

MPS IVA, also known as Morquio A syndrome, is caused by pathogenic variants in the gene encoding the enzyme N-acetylgalactosamine-6-sulfate sulfatase (GALNS). The enzyme encoded by the defective gene is dysfunctional, causing intracellular accumulation of chondroitin-6-sulfate (C6S) and keratan sulfate (KS) in several tissues, particularly in bone, cartilage and cornea [6]. The result is a systemic skeletal chondrodysplasia, the typical clinical manifestation of MPS IVA [7]. More than 180 pathogenic variants in the GALNS gene have been identified, accounting for the large variety of MPS IVA phenotypes observed [8]. Even though the musculoskeletal system is the most significantly involved, multiple clinical presentations are observed, thus MPS IVA patients may require multi-disciplinary approaches for diagnosis and management [9]. Also, as Morquio A is the MPS with the longest overall survival, it often requires transfer of care from pediatric to adult physicians, arising the issue of appropriate transition. Clinical manifestations vary from a “classical” syndrome, characterized by early-onset and rapid progression of severe systemic bone dysplasia, to a slowly progressive later-onset (mild) form, with less severe bone involvement. An intermediate form has also been described. The “classical” phenotype is largely prevalent, accounting for 68.4% of all individuals affected with MPS IVA in the International Morquio Registry, while only 9.8% were categorized as mild and 15.1% as intermediate [10]. The severity of symptoms is determined by the degree of skeletal and joints’ involvement. The most typical manifestation in MPS IVA affected patients is short stature, accompanied by spinal cord compression, spinal instability and thoracolumbar kyphoscoliosis, *genu valgum*, joint hypermobility, hip subluxation and dysplasia [11]. Spinal cord compression is the leading cause of mortality for MPS IVA patients, with an average life expectancy of 20–30 years if left untreated [12].

Traditionally, patients with MPS IVA have been managed exclusively by supportive measures, including symptom-based medical management, physical therapy, rehabilitation and surgery, but management options expanded in recent years [9]. Enzyme replacement therapy for MPS IVA using recombinant human GALNS, or elosulfase alfa [13], was approved by FDA and EMA in 2014, for children and adults with Morquio A syndrome [14]. In clinical trials performed in children and adults with MPS IVA, weekly intravenous administration of elosulfase alfa provided significant and sustained improvements in urinary levels of KS [15], with significative progresses in mobility and endurance scores (6 min’ walk test (6MWT)). Data were further confirmed in long term studies, also showing improved pulmonary function and activities of daily living (ADL) [16, 17]. Overall, these findings suggest that long-term elosulfase alpha ERT is well tolerated and associated with partial recovery of the functional status, improving Morquio A patients’ ability to perform ADL [18]. As a result, the use of elosulfase alfa is recommended in the last European Guidelines for the management of MPS IVA [19].

With this publication we aim to outline the landscape of MPS in Southern and Eastern European Countries, with particular reference to MPS IVA management, to highlight critical features in general management of disease and describe ERT availability.

## Methods

**Fig. 1:**
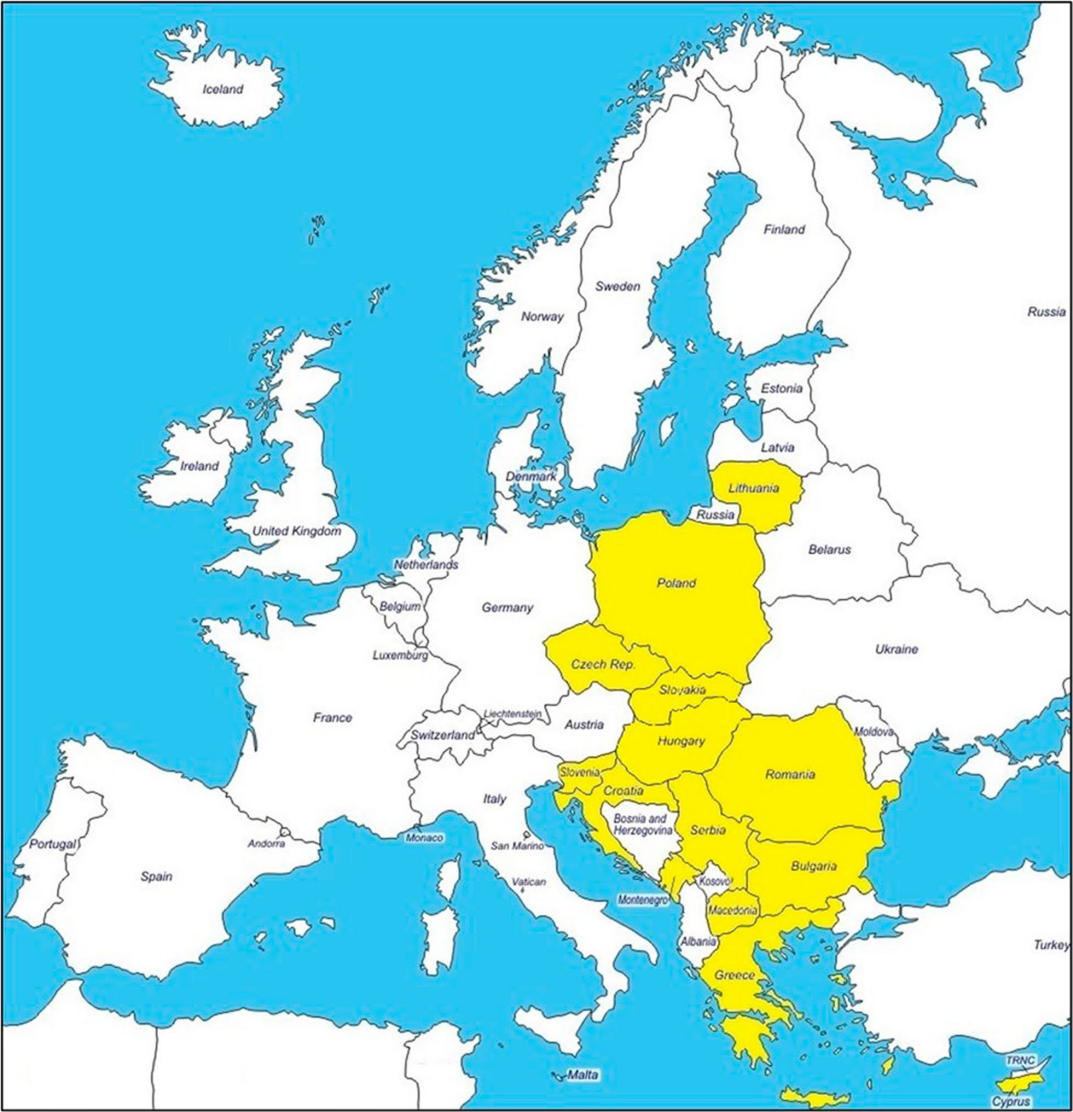
South and Eastern European countries involved in the “Mucopolysaccharidosis Management Physician Survey”, in yellow

The "Mucopolysaccharidosis Management Physician Survey" was conducted online—via the SurveyMonkey platform (www.surveymonkey.com)—between March and June 2020. Participants were recruited via e-mail, sending them a personalized link to access the survey on the SurveyMonkey platform. The questionnaire was developed by the scientific coordinator of the project based on particular expertise in management of MPS patients. 19 MPS Experts from 14 different Eastern and Southern European countries participated in the survey (Bulgaria, Croatia, Cyprus, Czech Republic, Greece, Hungary, Lithuania, Montenegro, Poland, Republic of North Macedonia, Romania, Serbia, Slovakia, Slovenia) (Fig. 1). In the second phase of the project, in May 2021, the experts met in a Virtual Working Group Meeting, together with the scientific coordinator, to analyze the results of the first Survey. 16 experts in the management of MPS patients from 13 Eastern and Southern European countries participated to the meeting (Bulgaria, Croatia, Cyprus, Czech Republic, Greece, Hungary, Lithuania, Montenegro, Poland, Republic of North Macedonia, Romania, Serbia, Slovakia). 4 experts who contributed to the first survey could not participate to the meeting because of organizational issues and retirement, and 1 expert from Poland only participated to the second survey. During the meeting, results were discussed and some issues for further investigation were identified. Thus, a series of questions were included in a second questionnaire to be sent to this group of experts to refine data obtained from the first survey. The second "Mucopolysaccharidosis Management Physician Survey" was sent to this group of experts with the same modality between May and June 2021.

The Surveys consisted of a series of mostly closed questions, logically grouped into the following areas:

1. Screening: country of practice of physicians and main specialty
2. Physician Characteristics and Clinical Practice: demographic characteristics of MPS patients in each respondent’s clinical practice
3. Experience with MPS type IVA (MPS IVA, Morquio A syndrome)
4. Management and Treatment of MPS type IVA: local practice in overall management, treatment and outcome assessment of patients with MPS IVA.

## Results

These results are based on retrospective data collected during two rounds of the "Mucopolysaccharidosis Management Physician Survey". Overall, 19 experts in the management of MPS from 14 Southern and Eastern European countries participated to the first surveys (Fig. 1) and 16 of them, from 13 countries, also responded to the second. Combined results from the first and the second survey are presented.

### Country of practice of physicians and main specialty

19 physicians with expertise in MPS management from 19 reference centers, distributed across 14 different European countries, participated in the first survey—2 centers in Bulgaria, 2 in Croatia, 1 in Cyprus, 1 in Czech Republic, 2 in Greece, 1 Hungary, 1 Lithuania, 1 in Montenegro, 1 in Poland, 1 in the Republic of North Macedonia, 1 in Romania, 3 in Serbia, 1 in Slovakia and 1 in Slovenia. Due to retirement of some physicians and other organizational issues, 2 centers from Serbia, 1 from Slovenia and 1 from Bulgaria did not participate to the second survey. One center from Poland that was not present during the first survey, participated to the second. Thus, data collected in the second round describe the real-life of MPS for 16 centers in 13 Eastern and Southern European countries—1 center in Bulgaria, 2 in Croatia, 1 in Cyprus, 1 in Czech Republic, 2 in Greece, 1 Hungary, 1 Lithuania, 1 in Montenegro, 2 in Poland, 1 the Republic of North Macedonia, 1 in Romania, 1 in Serbia, 1 in Slovakia.

**Fig. 2:**
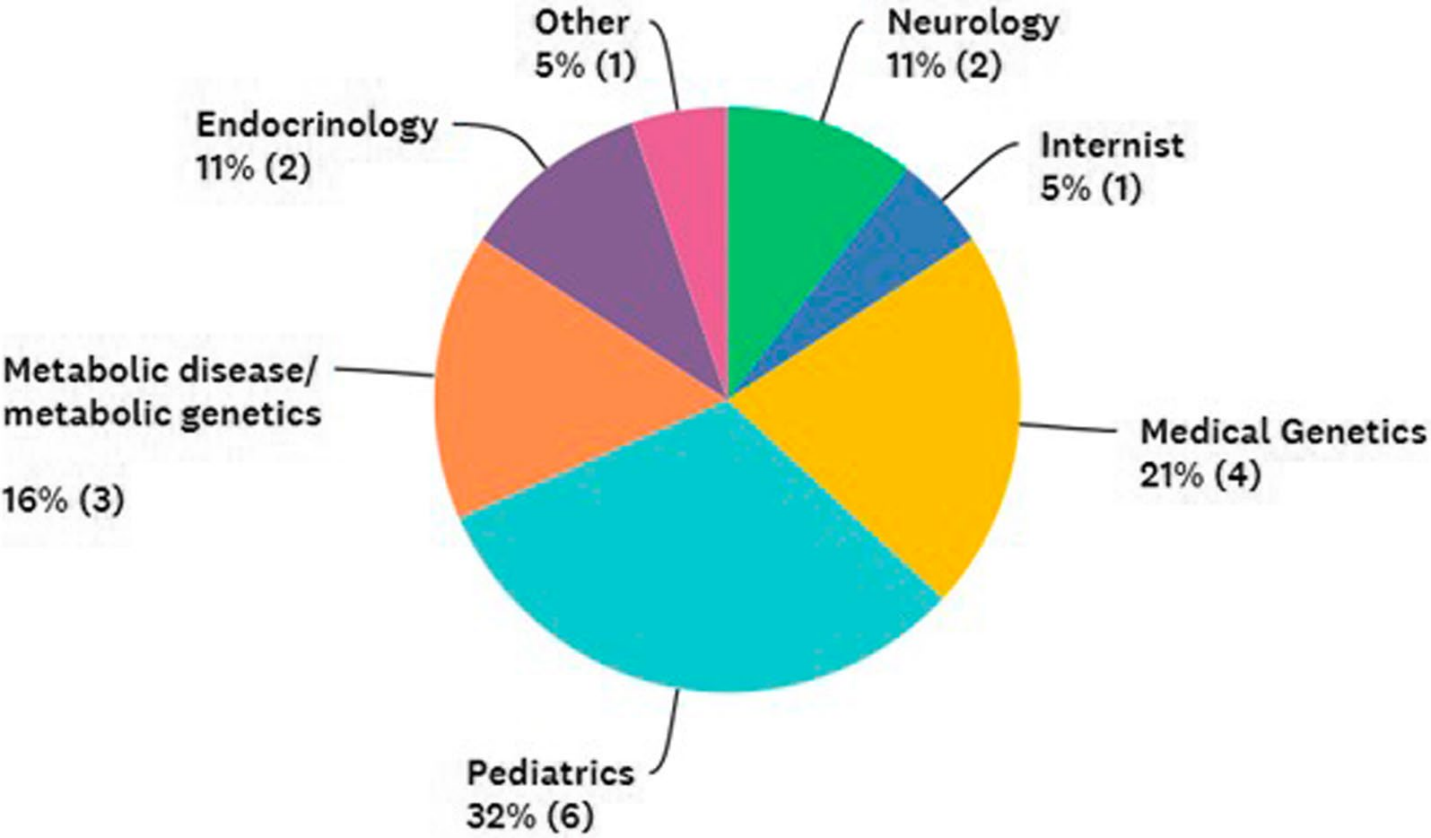
Physicians’ main specialty as reported after the first survey. In parentheses the absolute number of specialists

Overall, among 19 physicians responding in the first survey, 53% were pediatrician or medical geneticists (Fig. 2).

In the second survey, the specialty of physicians was defined in more detail. Only 19% of physicians (3/16) taking care of MPS patients were adult metabolic specialists, while 44% (6/16) were pediatrician. 44% of physicians were geneticists, routinely treating both pediatric and adult patients. Only one center specialized exclusively in pediatric care, but they also follow up patients during adulthood. 50% of participants answered that they normally take care of both pediatric and adult patients, while 19% confirmed to manage only adults and 31% only children.

Concerning the collaboration with an adult center to refer the patient after childhood, almost 38% of respondents confirmed the collaboration, while 2 physicians (12%) declared to have no collaboration with an adult center. Three experts commented that there is no official dedicated center for the management of adult patients with inherited metabolic disease in their countries, but there is a high level of collaboration between pediatricians and adult specialists. One expert, with a specialization in pediatric and genetics, affirmed that in his center MPS patients are managed by pediatricians throughout their life.

Overall, these data suggest the lack of a structured process of transition of care, with the majority of MPS patients remaining in the hands of pediatricians throughout transition and in adulthood. Only a minor percentage of specialists taking care of adult MPS patients are specialized physicians for adults.

Concerning the expertise of physicians, about 50% of physicians had practiced medicine post residency for more than 25 years, all of them in an academic setting.

### Patients with a confirmed diagnosis of MPS included in the study

Almost all the centers (95%) declared to have managed at least one patient potentially affected by MPS IVA.

**Fig. 3:**
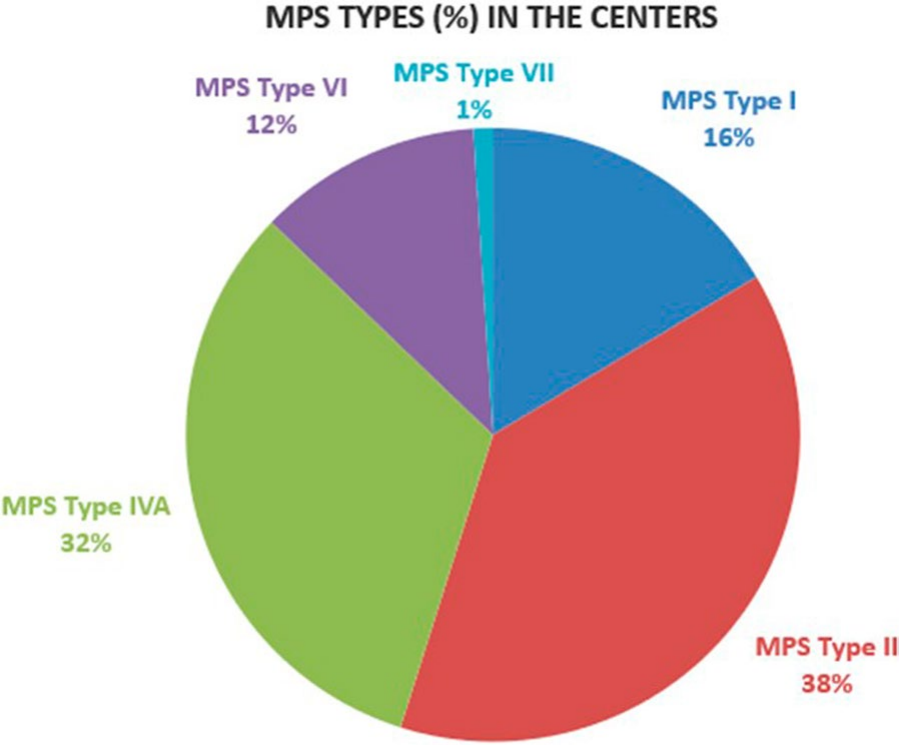
Relative frequency (%) of MPS types in the population of patients from all centers

In the second survey, the 16 experts reported that they managed in total 195 patients with a confirmed diagnosis of MPS I, II, IVA, VI and VII. The most frequent MPS type was MPS II (38%), closely followed by MPS IVA (32%) (Fig. 3).

Among all patients with a confirmed diagnosis of MPS, 63% were pediatric patients and 37% adults, a particular high number, indicating an optimal management of MPS patients in the centers, despite the lack of a structured transition process.

Interestingly, MPS IVA was the most frequent MPS type in adult patients, 40% of all adults, while the largest group of pediatric patients was the MPS II group, 49% of all MPS affected children. Only 2 patients with a confirmed diagnosis of MPS VII were reported by 2 centers, both adults, not surprising considering the rarity of this MPS (Table 1).

**Table 1:** Number of patients of different age groups and MPS types

	0–5 years	6–12 years	13–18 years	> 18 years	Total
MPS Type I	9(4)	6(3)	5(3)	12(7)	32
MPS Type II	14(6)	31(8)	15(5)	15(7)	75
MPS Type IVA	0	17(8)	17(7)	29(7)	63
MPS Type VI	0	4(3)	5(4)	14(5)	23
MPS Type VII	0	0	0	2(2)	2
Total	23	58	42	72	195

In parentheses the number of centers managing these patients

### Patients on enzyme replacement therapy in different age groups

Participants were asked to report about the number of patients receiving ERT in their centers, specifying the type of MPS and the age group of patients (Table 2).

**Table 2:** Patients receiving ERT divided by MPS type and age group

	0–5 years	6–12 years	13–18 years	> 18 years	Total
MPS Type I	5(2)	2(1)	3(2)	11(6)	21
MPS Type II	9(4)	24(8)	12(4)	7(5)	52
MPS Type IVA	0	11(7)	8(5)	6(3)	25
MPS Type VI	0	3(2)	3(3)	8(5)	14
MPS Type VII	0	0	0	0	0
Total	14	40	26	32	112

In parentheses the number of centers managing the patients

In total, 57% of all MPS patients are on treatment with enzyme replacement therapy; among them, 71% are pediatric patients and 29% are adults. These data reveal the necessity to make ERT more available for adult patients and implement treatment.

The most numerous group among pediatric patients were children aged 6–12 years, 47% of total pediatrics (Table 1), mostly MPS II patients (53% of the age group) and MPS IVA patients (29%). Overall, 69% of patients in this age group were receiving ERT, among which 77% of MPS II patients in the age group and 65% of MPS IVA.

In the age group 13–18 years, the second most numerous among pediatric patients, 62% of patients were on ERT. Most patients have MPS IVA (40%) and 47% of them were receiving ERT. Among the age group, MPS II patients are the second largest and 80% are on ERT. Other MPS types are significantly less represented in this age group, though most of them are receiving ERT (60% of MPS I and MPS VI patients) (Tables 1 and 2).

In the age group 0–5 years, 61% of patients are receiving ERT and most of them are MPS II patients. Almost one third of patients in the age group have MPS I and 55% of them are on ERT. Actually, we don’t have data on HSCT for these patients, thus ERT could have not been administered in transplanted patients. Interestingly, no MPS IVA, VI or MPS VII patients on ERT were reported in this age group, suggesting the need of increasing awareness on early diagnosis for these MPS types.

In general, ERT is much more available for pediatric patients (65% of all affected children) than for adults (44% of adult patients with all MPS types receive ERT) (Table 2). Remarkably, although the MPS IVA group is the most numerous in the age group > 18 years, only 21% of them were reported to be on ERT treatment, maybe due to hurdles in regulatory or reimbursement procedures for having this patients treated. On the other hand, almost all adult patients with MPS I were receiving ERT and 55% of adults with MPS II. No patients with MPS VII were reported to be on ERT treatment.

**Fig. 4:**
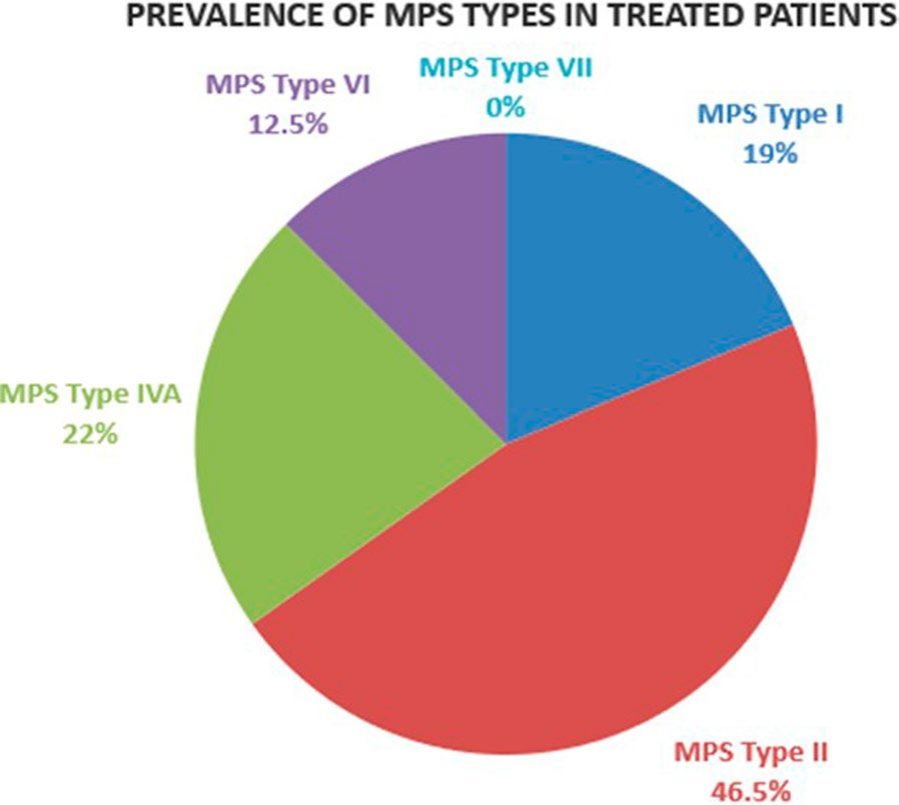
Relative frequency (%) of MPS types in the population of ERT treated patients

Overall, in patients of all ages, MPS II patients represent the majority of ERT treated individuals (46.5% of all treated patients), followed by MPS IVA patients (22%) and MPS I (19%). Only 12.5% of treated patients have MPS VI and no patient in the MPS VII group is on treatment (Fig. 4).

### Availability of ERT in different countries

Physicians were asked to report on the availability of ERT in their country, specifying the type of MPS treated patients (Table 3).

**Table 3:** Availability of ERT for MPS diseases in different countries

	Not available	Available for all ages	Available only for pediatric patients	Available only for some patients	Total
MPS Type I	2 (14%)	10 (71%)	1 (7%)	1 (7%)	14
MPS Type II	0 (0%)	12 (86%)	1 (7%)	1 (7%)	14
MPS Type IVA	2 (12.5%)	10 (62.5%)	2 (12.5%)	2 (12.5%)	16
MPS Type VI	2 (17%)	9 (75%)	0 (0%)	1 (8%)	12
MPS Type VII	9 (75%)	2 (17)%	0 (0%)	1 (8%)	12

In brackets the % of respondents

Overall, ERT availability is adequate for patients of all ages for all MPS types (accessible in 63% to 86% of centers) except for MPS VII patients, where it is available only in 17% of centers (Table 3). However, the rarity of MPS VII could explain the data. For MPS I, ERT is available for all age groups in 71% of centers and only 2 centers declared to have no access to ERT. Concerning MPS II patients, all centers that responded declared they have access to ERT. Generally, ERT is largely available for MPS II patients of all ages (86% of reference centers) and only for pediatric patients in one center.

ERT is accessible for patients with MPS IVA of all ages in 62.5% of specialistic centers. Importantly, 2 centers are able only to treat pediatric patients and in other 2 centers ERT is available only for some patients. 2 centers have no access to ERT for MPS IVA patients. These data arise the need of implementing ERT treatment for MPS IVA patients of all ages.

For MPS VI patients, ERT is largely available for all ages in most reference centers. In 1 center it is available only for some patients and in 2 centers it is not available.

### Difficulties in treating MPS IVA patients with ERT

In the first survey, 84% of experts from 19 centers declared that ERT is available in their countries. In 69% of centers reimbursement is by individual patient request to the National Health Insurance Fund and in 31% by positive list, meaning national reimbursement.

However, in the second survey, most centers declared that it is a difficult and long process to get reimbursement for ERT. 18% of centers invoked organizational problems in performing follow up examinations and 12% organizational problems in performing ERT (Table 4).

**Table 4:** Reasons for difficulties in treating MPS IV patients

Answer choices	Responses
Difficult and long administrative process to get reimbursement	12 (75%)
Patients do not want to be treated	1 (6%)
Organization problems in performing ERT	2 (12%)
Organization problems in performing follow up examinations	3 (18%)
Others	4 (25%)

In brackets the percentage of responders

Some participants mentioned other reasons for difficulties in treating patients with ERT. For instance, one expert stated that ERT reimbursement is difficult because of frequent reports to the health insurance company about the effectiveness of treatment (every 6–12 months), for having allowance to continue treatment. Another participant declared that ERT for MPS IVA is available in the country but not reimbursed.

Moreover, the average hours to perform application for reimbursement varies from 2 h to several days (not including the time to get a response from the healthcare authorities), considerably limiting ERT implementation.

### Defining the group of MPS IVA patients

MPS IVA patients represent the second largest group of all MPS patients included in the study (32%). 54% are pediatric patients and 46% are adults (Table 1). Interestingly, MPS IVA patients are the most numerous among adults with all MPSs, maybe due to the high awareness on this type of MPS among reference centers included in the study. Most patients are in the age group 6–18 years (54%, equally distributed among 6–12 yrs and 13–18 yrs children) (Table 1). There are no MPS IVA patients in the 0–5 year age group, revealing the need for implementing a protocol to enable early diagnosis in this patient population. Overall, 40% of MPS IVA patients of all ages are on ERT treatment in the 16 centers involved in the study. 6 sibling pairs are included in the group.

Among patients with MPS IVA who are on ERT treatment, only 4 were reported to be wheelchair bound (Table 5).

**Table 5:** Wheelchair bound and ambulatory patients with MPS IVA on ERT treatment

	0–5 years	6–12 years	13–18 years	> 18 years	Total
Wheelchair bound	0	0	2	2	4
Ambulatory	0	10	6	4	20
Total	0	10	8	6	24

Almost all patients are defined as classical phenotypes, reflecting data from the International Morquio Registry [10], except 1 patient on ERT in the age group 13–18 years and 1 adult patient who is not treated.

Moreover, to better understand the prevalence of MPS IVA in the countries represented, experts were asked if they were aware of any other center in their country taking care of MPS IVA patients. Among 16 experts, only 6 answered they knew other centers taking care of patients with MPS IVA at the time of the survey. However, they were unable to provide the number of these patients.

### Methods to confirm diagnosis of MPS IVA before starting treatment

**Fig. 5:**
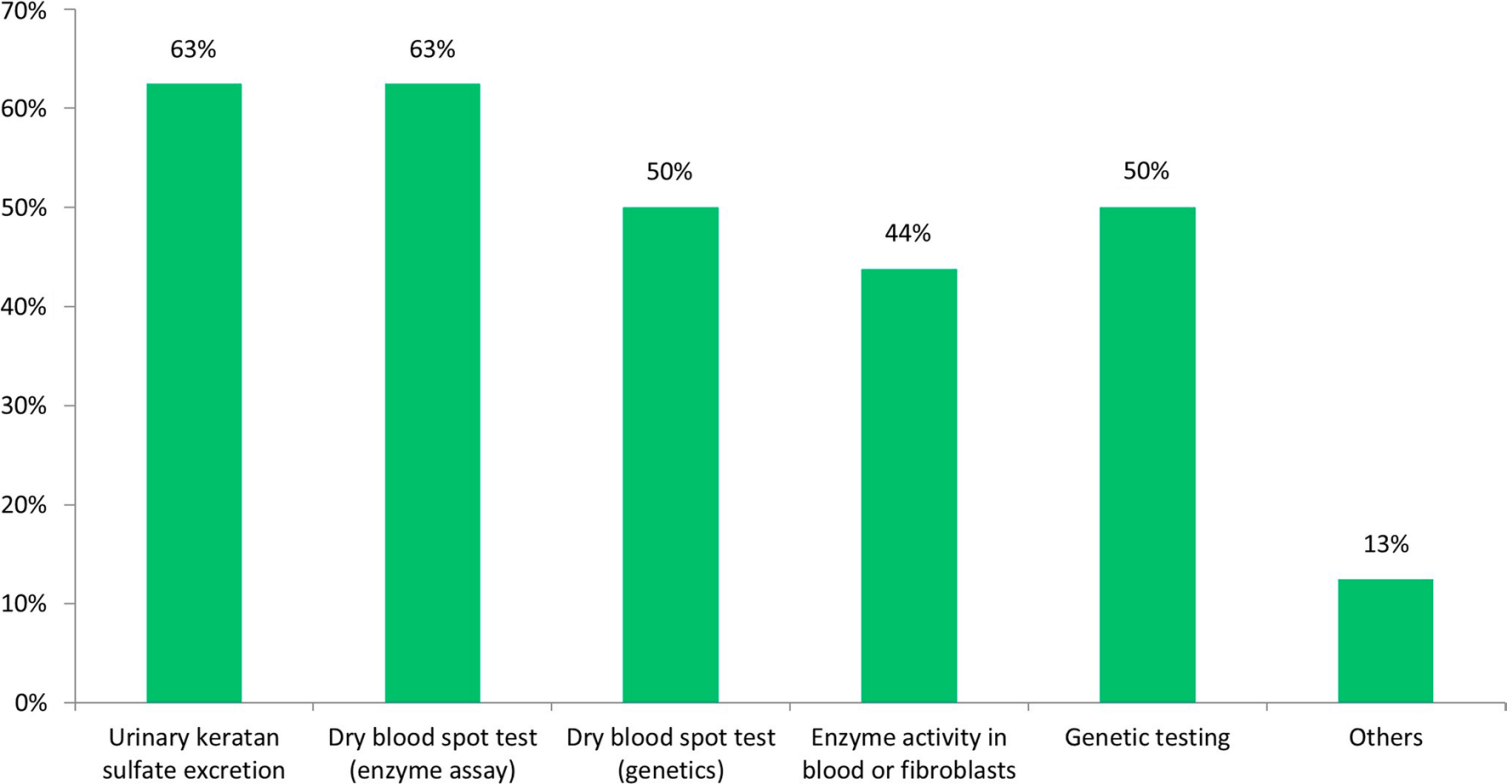
Appropriate methods to confirm diagnosis of MPS IVA before starting ERT treatment (% of respondents).

In the second survey, the experts were asked to define the methods currently used to confirm diagnosis of MPS IVA before initiating ERT treatment (Fig. 5).

The most cited methods were urinary KS and dry blood spot test for enzyme assay, followed by genetic testing and dry blood test for genetics, that were considered appropriated by 50% of respondents. 7 experts mentioned enzyme activity test (in blood or fibroblasts) and 2 declared to use other methods.

### Routinely performed tests in MPS IVA patients

In the first survey, participants were invited to describe the clinical practice for routine monitoring of MPS IVA patients. In particular, experts were asked to detail the assessments performed in their centers before initiating ERT and for monitoring response to treatment.

Most experts (84%) declared to routinely perform endurance test (6MWT) before introducing ERT. Endurance testing is carried out in most cases (84%) every 6–12 months. There is no complete agreement on the most appropriate interval for monitoring ERT response using 6MWT. A slight majority of experts (53%) fixes the interval at 6 months, 32% at one year, 10% at 3 months and 5% at 18 months.

Respiratory function is also routinely performed before initiating treatment by 89% of experts. Concerning time schedule for assessment, protocols are not widely agreed, maybe due to different facilities for having patients tested in different countries. More than 50% of specialists involved perform respiratory function at least annually, 37% every 6 months, while 10% of experts never perform the test.

**Fig. 6:**
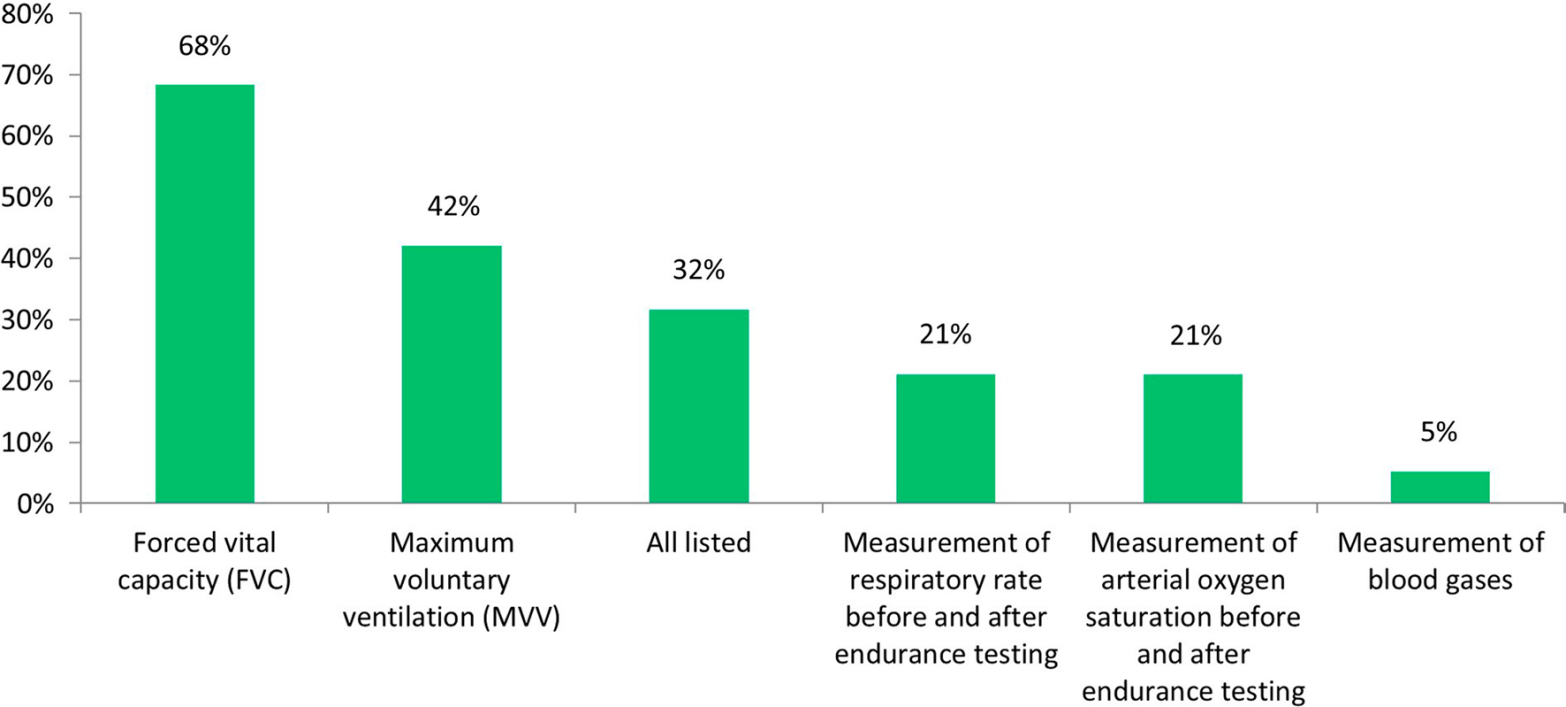
Respiratory function parameters considered appropriate to demonstrate the clinical benefit of ERT in patients with MPS IVA (% of respondents)

The parameter of respiratory function considered most appropriate to demonstrate the clinical benefit of ERT is Forced Vital Capacity (FVC) for 68% of experts, followed by Maximum Voluntary Ventilation measurement (MVV) (42%), while 32% of respondents believe that all parameters indicated in the question are appropriate (Fig. 6).

**Fig. 7:**
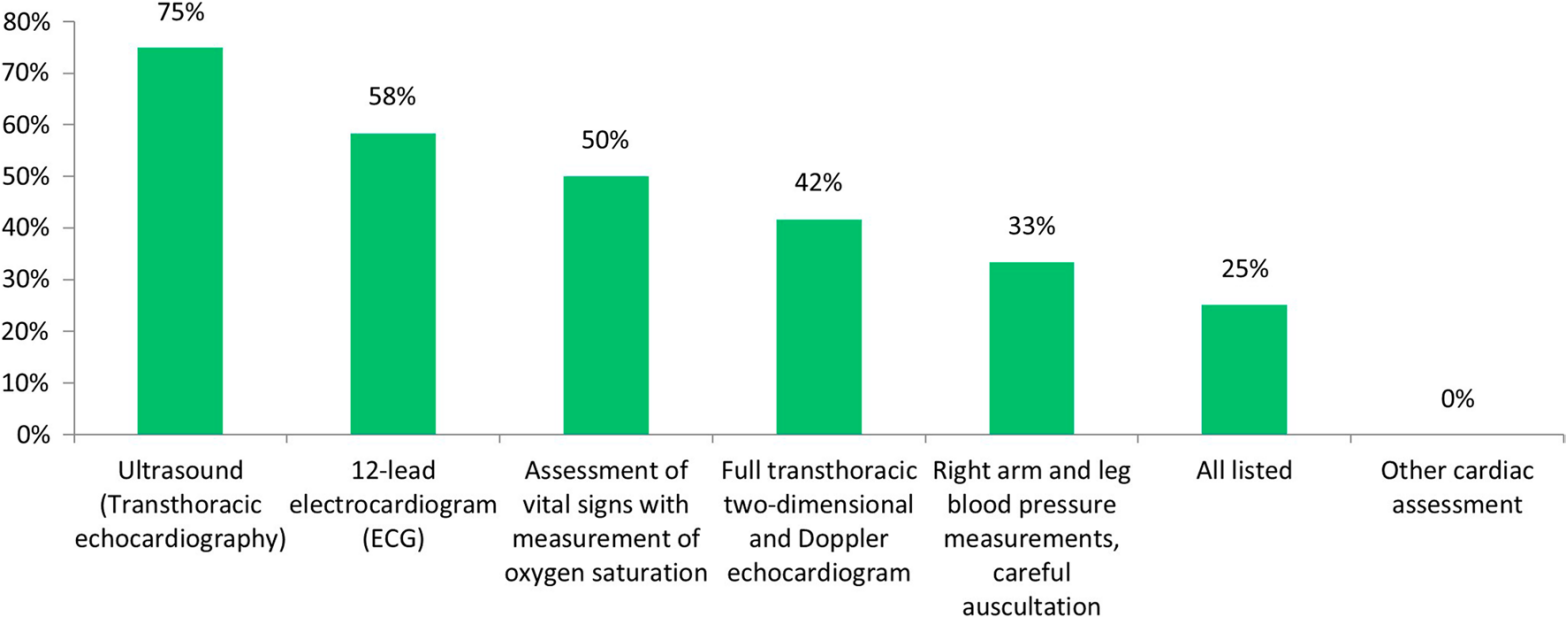
Cardiac function parameters considered appropriate to demonstrate the clinical benefit of ERT in patients with MPS IVA (% of respondents)

Cardiac function assessments were defined appropriate measures to prove clinical benefit of ERT by 68% of experts. Among cardiac function parameters, ultrasound was considered the most appropriate, followed by 12-lead electrocardiogram and vital sign measurement (Fig. 7).

### Evaluation of important outcomes for monitoring response to ERT in MPS IVA patients and difficulties in following the guidelines for MPS IVA completely

In the first survey, 89% of experts declared to follow the recommendations in the last published European guidelines [19] for monitoring the effectiveness of ERT in MPS IVA patients. However, only 32% of experts affirmed to have a clear definition for “satisfactory or non-satisfactory” response to ERT and most of them (58%) answered they could not give a definitive response. Thus, to better understand the real life clinical practice for ERT evaluation at a regional level, in the second survey participants were asked to estimate the importance of assessments for monitoring the effectiveness of ERT in MPS IVA patients recommended in the European Guidelines, scoring them as “very important” or “important”, “somehow important” or “not important”. Experts were also asked to define the availability of assessments in their countries and how difficult are to perform, scoring all examinations as “very easy” or “easy”, “somehow difficult”, “difficult” or “very difficult”, (Table 6).

**Table 6:** Availability of assessments and difficulties in following the guidelines for MPS IVA completely compared to self-evaluation of important outcomes for monitoring response to ERT in MPS IVA patients

	Availability of assessment					Self-evaluation of importance				
	Very easy	Easy	Somehow difficult	Difficult	Very difficult	Very important	Important	Somehow important	Not important	Total
General Physical examination	**50%**	**31**%	19%	0%	0%	**88%**	**12%**	0%	0%	16 (100%)
Neurological examination	**37%**	**44%**	19%	0%	0%	**44%**	**37%**	19%	0%	16 (100%)
Joint range of motion (JROM)	*25%*	*50%*	19%	6%	0%	**44%**	**50%**	0%	6%	16 (100%)
Growth	**62%**	**25%**	13%	0%	0%	**44%**	**50%**	6%	0%	16 (100%)
Endurance (6MWT)	*50%*	*25%*	25%	0%	0%	**56%**	**31%**	13%	0%	16 (100%)
X-rays	**56%**	**31%**	13%	0%	0%	**19%**	**69%**	12%	0%	16 (100%)
MRI	*25%*	*44%*	19%	12%	0%	*44%*	*31%*	19%	6%	16 (100%)
CT scan	*31%*	*44%*	19%	6%	0%	*25%*	*38%*	31%	6%	16 (100%)
Cardiology (echo, ecg)	**44%**	**44%**	6%	6%	0%	**31%**	**69%**	0%	0%	16 (100%)
Respiratory function (MVV, FVC)	*31%*	*38%*	31%	0%	0%	**56%**	**44%**	0%	0%	16 (100%)
Sleep study	***0%***	***31%***	50%	6%	13%	*13%*	*56%*	31%	0%	16 (100%)
Oral health	*31%*	*31%*	38%	0%	0%	*6%*	*50%*	44%	0%	16 (100%)
Eye examination	*37.5%*	*37,5%*	25%	0%	0%					
Ear examination	**37%**	**44%**	19%	0%	0%					
Disease burden (EQ-5D-5L, MPS HAQ, PRO)	*19%*	*50%*	25%	6%	0%	**31%**	**56%**	13%	0%	16 (100%)
Anesthesia	*25%*	*31%*	25%	13%	6%	**44%**	**37%**	13%	6%	16 (100%)
Surgical interventions	***13%***	***31%***	37%	13%	6%	**44%**	**37%**	13%	6%	16 (100%)
Electrophysiology	*19%*	*38%*	31%	6%	6%	**19%**	**62%**	6%	13%	16 (100%)
Lab testing	**32%**	**56%**	6%	6%	0%					

In bold assessments defined as “very easy or easy” or “very important or important” by 80–100% of experts. In italic assessments defined as “very easy or easy” or “very important or important” by 50–79% of experts. In bolditalic assessments defined as “very easy or easy” or “very important or important” by < 50% of experts. *6MWT* 6 min walk test; *CT scan* computerized tomography scan; *ECG* electrocardiogram; *echo* echocardiogram; *EQ-5D-5L* 5-level EuroQoL -5D questionnaire; *FVC* Forced Vital Capacity; *JROM* Joint Range of Motion; *MPS-HAQ* MPS health assessment questionnaire; *MRI* Magnetic Resonance Imaging; *MVV* Maximum voluntary ventilation; *PRO* patient reported outcomes. In parentheses the % of respondents

Actually, although most experts (> 50%) defined assessments for monitoring ERT recommended in guidelines as important, several difficulties in performing some examinations emerged. Particularly, all experts consider general physical examination, cardiology assessments and respiratory function as necessary outcomes for monitoring ERT efficacy. However, general physical examination is difficult to perform in almost 20% of centers and respiratory function in almost one third of centers. Organizational hurdles were invoked by some experts as the cause of difficulties in routinely perform even basic assessments.

Notably, surgery and sleep studies, although scored as important by most of participants (81% and 69% respectively), are difficult in many centers. Sleep studies, in particular, are not available in most centers (69%), maybe due to lack of facilities or qualified specialists. Surgery was defined difficult in more than 50% of the centers. All other assessments for follow-up of MPS IVA patients are available in more than 50% of centers participating in the study. Overall, large availability of the assessment (> 80% of centers) mostly matches evaluation of importance for general physical examination, growth assessment, neurological examination, X-Ray and cardiology. On the other hand, other examinations considered important by the large majority of experts—i.e. JROM, endurance, respiratory function, disease burden indexes, anesthesia and electrophysiology—should be implemented in some centers.

## Discussion

This survey investigated the real life MPS management in Southern and Eastern European countries. Data were collected between March 2020 and June 2021, during the Covid-19 pandemic, thus there might be a bias due to the incidence of disease on patients with MPS followed by the centers or to restrictions to contain the pandemic that might have influenced follow-up visits or treatment schedules. Actually, changes in therapy regimen due to the pandemic were reported by 64.4% of centers in the recent survey conducted in 73 centers in the European Reference Network for Hereditary Metabolic Disorders (MetabERN), in 21 European Countries [20]. Outpatient visits were almost cancelled (75–100%) by more than 50% of specialists that responded to the MetabERN survey, to protect patients with inherited metabolic disorders (IMD) from Covid-19, considering the vulnerability of these subjects to Covid-19 complications, possibly causing degeneration of their disease. We might say that information on the influence of Covid-19 on patients with MPS in the region would certainly be worthy of consideration but not particularly relevant to our study, since we do not provide data on disease course of patients or on frequency of follow-up or treatment schedule. Thus, the present study should be read as a picture of MPS management in Southern and Eastern European Countries. However, it is important to underline that the aim was not to provide a snapshot of disease epidemiology and management in the region, but to understand the awareness about MPS management and treatment in specialists and in the health systems in general.

MPSs are very rare inherited disorders, characterized by early clinical symptoms, progressive course and involvement of virtually all organs and systems. Due to the progressive skeletal chondrodysplasia MPS IVA patients often require surgical interventions in the upper cervical spine, generally before the age of 10 years [10]. Surgery in the lower extremities is also performed in most patients, although orthopedic interventions generally fail to provide resolutive and long lasting benefits [21]. Involvement of other organ systems can also lead to significant morbidity, including respiratory compromise, obstructive sleep apnea, valvular heart disease, hearing impairment, micturition disorders, visual impairment from corneal clouding, dental abnormalities, and hepatomegaly [2, 22]. As a result, patients with MPS experience a low quality of life and require continuous help from other people all along their life. Generally, diagnosis is delayed until the appearance of first symptoms. However, considering the progressive nature of MPS disorders, treatment should be initiated as soon as possible after diagnosis, possibly even before evident clinical features are visible [23]. Considering these issues, this study aims to describe the landscape of disease management in Southern and Eastern Europe and to outline eventual gaps in diagnosis, follow-up, transition and treatment of patients with MPS. ERT is currently the most appropriate and effective treatment for lysosomal enzymatic disorders. Beyond the specificity of effects in different MPS types, generally ERT aims to reduce GAG accumulation, thus delaying progression of disease and ameliorating quality of life [24]. First introduced in 1991 for the treatment of Gaucher disease [25], in the last 20 years ERT has become available for other lysosomal storage disorders, including some types of MPS. Laronidase was the first ERT for MPS, approved for the treatment of MPS I (Hurler, Hurler-Scheie, Scheie syndrome) in 2003 [26]; subsequently the treatment with galsufase became available for MPS VI (Maroteaux-Lamy syndrome), approved by the European Medicines Agency (EMA) in January 2006 [27]. ERT with idursulfase for MPS II (Hunter syndrome) is available in Europe since January 2007 [28] and elosulfase alfa for MPS IVA (Morquio A syndrome) was approved by the FDA and EMA in 2014 [14]. More recently, in August 2018, ERT with vestronidase alfa also became available for the treatment of MPS VII (Sly syndrome) [29] [for review on ERT see reference 30]. Indications vary across different MPS, but generally ERT is used in patients with moderate to severe disease or complications, although data indicate that treatment is much more effective if started very early (preferably in the pre-symptomatic phase) [23, 24]. However, ERT is not effective in the neuropathic forms of MPS, given the inability of recombinant enzymes to cross the blood–brain barrier. Also, implementation of ERT varies across different countries, due to hurdles in national regulatory or reimbursement procedures [31]. To understand access to treatment with ERT for MPS, the difficulties of reimbursement, as well as the accessibility to follow-up examinations and assessments recommended in the relevant practice guidelines, we performed a survey analysis among physicians specialized in the treatment of patients with MPS in 14 Southern and Eastern European countries. The survey was distributed using SurveyMonkey, an online platform allowing easy accessibility to the questions for the physicians and fast collection of responses. The first SurveyMonkey included a series of general questions to understand fundamental aspects of MPS management in the region. Results revealed an interesting context of disease management, with a high number of MPS patients managed in the centers and a high level of care, despite regulatory and administrative hurdles. Thus, after discussing with all participants the results of this first survey, a second survey was performed, to better describe some critical aspects that had emerged, mainly concerning the age of patients with MPS included in the study and the availability of treatment and follow-up examinations. Due to the increasing availability of diagnostic methods and advances in medical and surgical treatment options in recent years, a higher number of patients are diagnosed at an earlier age, life expectancy has increased, and more patients reach adulthood. In this setting, the issue of transition of MPS patients from pediatric to adult care is of particular concern [5]. The management of transition in patients with inherited metabolic disorders has been recently investigated in a large multicenter European survey performed by the MetabERN network [32]. Although the MetabERN survey was referred to the management of all IMDs and not focused on MPS, 87.1% of respondents were physicians specialized in the management of lysosomal storage disorders. Comparing our data with those of the MetabERN survey, our study revealed a significantly higher number of adult specialists taking care of MPS patients, a smaller percentage of pediatricians and a largely greater proportion of clinical geneticists (Table 7).

**Table 7:** Specialization of physicians taking care of MPS patients (our survey) compared to results of the MetabERN survey on management of IMD in Europe [33]

	Our survey	MetabERN survey on IMD
Adult physicians	19%	11.1%
Pediatricians	44%	65.1%
Clinical geneticists	44%	4.8%
Physicians treating only adults	19%	6.4%
Physicians treating both children and adults	50%	84.2%
Collaboration with adult centers	38%	30.7%

This is particularly relevant if we consider that 37% of MPS patients included in our study were adults, an unexpectedly high number. Although it was not possible to find a comparison in the literature, numbers are certainly relevant and indicate the optimal management of patients with MPS in the centers, particularly considering the attention dedicated to adult patients. Also, despite regulatory difficulties in treating adult patients in many countries, almost half of adult patients in our study are receiving ERT (44% of total adults and 29% of all patients treated). These data outline a positive situation for MPS management in Southern and Eastern European countries included in the study. As it also has emerged during discussion within the group of experts, positive results are probably due to the high rate of physician engagement, in terms of willingness and personal involvement to provide the highest level of care for patients, in spite of hurdles in the healthcare system.

Describing the relative frequency of MPS types in the centers involved in our study, some peculiarities deserve consideration. Epidemiology of different types of MPS indicates that it may be related to regional and ethnic background. In Europe, MPS I, III and II are respectively the most prevalent, followed by MPS IV [4]. Similarly, MPS III is the most frequent in the US, MPS I and II come shortly after, while MPS IV is significantly less prevalent [33]. In our study, the most frequent MPS type was MPS II (39%), closely followed by MPS IVA (33%). MPS IVA affected individuals also represented the large majority of adult patients. No MPS III patients were included in our study because we focused on treatable MPS forms. A similar high incidence of MPS II was only described in Sweden by Malm and coll. [34], while comparable data for MPS IVA were found in Norway and Denmark in the same study. The higher frequency of MPS II and the high number of MPS IVA patients in our study could be probably explained by the higher knowledge of physicians of these types of disease, leading to higher diagnosis. The high number of MPS IVA adult patients in Southern and Eastern Europe could be similarly explained by the high awareness of this disease among physicians. This, together with a high level of care in the centers, would lead to a high rate of diagnosis for MPS IVA. We have no explanation for our data based on region or ethnic group specific MPS types, nevertheless MPS IVA prevalence in our study is particularly significant and needs to be further investigated.

Concerning the accessibility to ERT treatment for MPS patients, according to our survey ERT is largely available for patients of all ages for MPS I, II, IV and VI, while it is still not available for MPS VII patients in most countries. However, this is not surprising, considering the extreme rarity of MPS VII. Interestingly, no MPS IVA, VI and VII patients under the age of 5 years are on treatment in all centers, suggesting the need for implementing a protocol to enable early diagnosis, in order to initiate treatment as soon as possible. Unfortunately, ERT is available in all centers only for MPS II patients, which are also more frequently treated compared to patients with other MPS. Moreover, reimbursement for ERT is not accessible in all countries or the hurdles to overcome administrative issues are demanding. Mostly, the process to get reimbursement is complicated, excessively time-consuming and, in some countries, it is necessary to apply for reimbursement for each patient, thus time from prescription to treatment is often long. Also, some experts claimed a pressing process to have renewal of reimbursement based on effectiveness for each patient or declared difficulties in performing follow-up examinations to assess effectiveness. It seems that availability of ERT and reimbursement is a particular concern in the three non-EU countries involved in our study, where treatment is mostly available only for patients with MPS II and IVA and preferably for pediatric patients. Accordingly, numbers of patients on treatment from these countries are small if compared to those in EU countries analyzed in the survey, probably because of additional economical, regulatory or organizational barriers outside the EU.

Overall, our results are consistent with the survey performed by Heard in 2020 [31], describing the availability of orphan medicines in 18 European countries in the MetabERN Network, including ERT for MPS I, II, IVA and VI. Among 25 orphan medicines analyzed in the study only 5 are available for the whole patient population, 12 are delivered to one-half and 8 products are rarely delivered to patients. Among the products prescribed to almost all patients when accessible, are idursulfase for MPS II and galsulfase for MPS VI. The restricted accessibility to treatment and the lack of reimbursement in several countries is a main finding of the study. Also, price negotiation for drugs do not take place at the European level, but at the member state level, probably causing particular budget constraints in some countries [35]. Comparing our data on ERT delivery with the availability of orphan medicines in the MetabERN network [31], it seems that availability of treatment in South and Eastern European countries is consistent with average data in other European regions. Also, the percentage of MPS treated patients in our centers is remarkable, particularly if we refer to almost one-half of MPS adult patients receiving ERT in our study. Still, in spite of regulatory, organizational and economical barriers, patients with MPS in Southern and Eastern European countries are receiving high level treatment. However, any effort should be addressed to reduce hurdles to accessibility to treatment.

The availability of follow-up assessments has been particularly investigated in MPS IVA patients from the 16 specialized centers responding to the second survey. Among the assessments recommended in the European guidelines for MPS IVA patients [19], it seems that surgery, anesthesiology and sleep studies, although considered important outcomes for monitoring ERT efficacy by most participants, are difficult to perform in many centers. Even general physical examination, theoretically a routine assessments considered mandatory by all experts, is difficult to perform in 20% of centers. Also, surgical intervention, although necessary to reduce skeletal deformities in many patients and defined as important or very important by most experts, is difficult to perform in most centers involved in the study. Reasons indicated for difficulties were mostly organizational problems, invoked by some experts as the cause of difficulties in routinely perform even easy assessments, such as general physical examination. Administrative issue and financial problems in some centers were also mentioned. Also, experts reported the limited expertise about MPS and the inadequate number of qualified physicians and scientists with particular experience in management of MPS patients—in particular surgeons, anesthesiologists and neurologists with specific experience, who are able to meet the traditional needs of MPS patients. This is probably the explanation for problems in performing sleep studies, surgery and anesthesiology. Difficulties in collaborating with other specialists and the lack of a structured multidisciplinary management of patients was also claimed by some experts. This is not different from the situation of MPS management described in most other countries and the inadequate availability of specialist experts in MPS has also been claimed in other studies [36, 37]. Nevertheless, the matter is particularly relevant and needs to be addressed. The implementation of training for physicians and other HCPs involved in care of MPS patients is a main necessity, as well as increasing the awareness about disease, spreading the knowledge about guidelines and best practice and facilitate multidisciplinary collaboration.

### Limitations

The study has limitations which require consideration. This is a retrospective survey study of current practice for MPS management in 14 Southern and Eastern European countries, thus actual practice may vary from that reported. In addition, although main centers for disease management were included in the study, it does not capture all practice across the region, potentially introducing selection bias. Also, the study doesn’t include data on HSCT for MPS patients, particularly for MPS I, which could partially explain the lack of ERT for some patients. Despite these limitations, the survey describes the real life of MPS in the region for the first time and provides a valuable insight into current practice.

## Conclusions

The survey outlined the real life MPS management in Southern and Eastern European countries. The landscape of MPS in the region reveals a comparable picture to that of other European countries, as for the epidemiology of MPS types, treatment accessibility and follow-up difficulties. Still, in order to be able to start treatment as early as possible and make it available to more patients, diagnostic algorithms should be established and reimbursement for ERT should be simplified. In addition, this survey showed the need of further improving overall disease management and enhancing multidisciplinary collaboration among specialists, including through dedicated educational programs for specialists on surgical interventions, anesthesia and sleep studies.

## Data Availability

All data generated or analysed during this study are included in this published article.

## Abbreviations

6MWT: 6 Minutes walk test
ADL: Activities of daily living
C6S: Chondroitin-6-sulfate
CT scan: Computerized tomography scan
DBS: Dried blood spot
ECG: Electrocardiogram
echo: Echocardiogram
EMA: European Medicines Agency
EQ-5D-5L: 5-Level EuroQoL -5-dimensions questionnaire
ERT: Enzyme replacement therapy
FVC: Forced vital capacity
GAG: Glycosaminoglycans
GALNS: N-acetylgalactosamine-6-sulfate sulfatase
HSCT: Hematopoietic stem cell transplantation
IMD: Inherited metabolic disorder
JROM: Joint range of motion
KS: Keratan sulphate
LSD: Lysosomal storage disorder
MPS: Mucopolysaccharidoses
MPS-HAQ: MPS health assessment questionnaire
MRI: Magnetic resonance imaging
MVV: Maximum voluntary ventilation
PRO: Patient reported outcomes
